# Treating refractory depression in Parkinson’s disease: a meta-analysis of transcranial magnetic stimulation

**DOI:** 10.1186/s40035-018-0113-0

**Published:** 2018-03-22

**Authors:** Alexandra M. Lesenskyj, Megan P. Samples, Jill M. Farmer, Christina R. Maxwell

**Affiliations:** 1 Rowan University School of Osteopathic Medicine, Stratford, NJ 08048 USA; 2 Drexel Neurosciences Institute, 245 N 15th Street ms 423, Philadelphia, PA 19102 USA

**Keywords:** Parkinson’s disease, Depression, Transcranial magnetic stimulation

## Abstract

**Background:**

Parkinson’s disease (PD) is often accompanied by clinically identified depression. Providing effective pharmacotherapies that concomitantly treat both motor and psychological symptoms can pose a challenge to physicians. For this reason, alternatives to standard anti-depressant treatments, such as repetitive transcranial magnetic stimulation (rTMS), have been evaluated within the Parkinson’s population.

**Methods:**

A literature search was conducted on the PubMed database for all studies that evaluated rTMS as a treatment in patients with both depression and PD. A meta-analysis was performed on all studies that reported mean pre- and post-rTMS depression inventory scores. Widely used depression inventories included both self-report and clinician-administered measures. Effect size for individual study groups and across all studies was calculated.

**Results:**

Six of 7 studies meeting inclusion criteria reported significantly improved depression scores, large effect sizes, and significant *p*-values. Total weighted average effect size was calculated at 1.32 across all study groups that applied rTMS.

**Conclusions:**

Across all but one study, rTMS appears to effectively reduce depression scores among self-reported and clinician administered inventories. The total weight average effect size showed that, when considering study sample sizes and degree of findings, this form of neurostimulation can relieve PD patients of their depressive symptoms. Further, rTMS is a promising alternative to traditional anti-depressant therapies when treating refractory depression in patients with PD.

## Background

The prevalence of depression in Parkinson’s disease (PD) is underestimated at 40 % [1]. The comorbidity between PD and depression requires a careful balance of multiple pharmacotherapies [2]. Shared pathophysiology between the two conditions poses a challenge with respect to treating both motor and non-motor symptoms effectively. Dopamine is a common target in the treatment of both depression and Parkinson’s disease, often complicating effective management of symptoms [3]. Treatment is additionally complicated because this relationship occurs across a wide range of patients with varying responsiveness to typical anti-depressive medications [4]. For this reason, several alternative anti-depressant therapies have been explored for the treatment of depression in the PD population [5–8].

One novel treatment for depression in the general population is transcranial magnetic stimulation (TMS). This neurostimulative technique utilizes non-invasive coils that produce magnetic fields and currents that in turn affect neurons within specific cortical areas [9]. In particular, TMS to the left dorsolateral prefrontal cortex (DLPFC) and dorsomedial prefrontal cortex have been identified as viable treatment options [8, 10–12]. Clinical trials have examined both psychological and physiological changes that occur in correlation with TMS. These studies consider several depression inventories as well as electrochemical changes occurring in depressed patients subsequent to TMS.

Several widely accepted depression surveys have been used to demonstrate change in depressive symptoms when evaluating TMS [7, 8, 13]. In a sham-controlled randomized trial, George and colleagues used the Hamilton Depression Rating Scale (HAM-D), a clinician-administered tool, as a means to show that daily high frequency repetitive TMS (rTMS) to the left DLPFC yields high rates of remission as compared to sham stimulation [7]. Another multi-site trial used the Montgomery-Asberg Depression Rating Scale (MADRS) as a primary measure to show that TMS was more effective than sham treatment; in this study, the MADRS as well as the 17 and 24 item HAM-D were significantly improved at 4 and 6 weeks [8]. A self-report measure, the Beck Depression Inventory (BDI), has also been used to show remission in patients with depression who have been treated with this neurostimulation [13].

In addition to the psychological changes, physiological changes have been observed in patients with depression who were treated with TMS [14]. Functional connectivity of depressed patients may be made stable when certain phenotypes are treated with stimulation to the left DLPFC [15, 16]. Those patients who respond to high frequency stimulation show initially high functional connectivity in various brain regions that may indicate longstanding changes in connectivity associated with improved depression scores [17].

Success with TMS when treating depression raises the question of whether this method will be safe and effective in the Parkinson’s disease (PD) population. TMS has been assessed in the treatment of motor symptoms in PD [18]. These studies attest to the safety of this method and moderate ability to improve tremors [19, 20]. Recent research has begun to evaluate TMS for non-motor symptoms in PD patients [21]. Several studies of varying quality and design suggest that TMS is a promising alternative therapy to typical anti-depressant medications used for patients with PD [22–27]. This meta-analysis seeks to quantitatively and qualitatively evaluate the findings of these studies. This review aims to determine the relative efficacy of TMS as a treatment for clinical depression in PD patients.

## Methods

### Search criteria

A literature search was completed on the PubMed research database for key words transcranial magnetic stimulation (TMS), Parkinson’s disease, and depression. Other search terms did not increase the number of studies found. No time restrictions were used in the search.

### Inclusion criteria

Inclusion criteria required a study population of patients diagnosed with Parkinson’s disease, concomitant clinically identified depression, and treatment via TMS. Research articles were only included if pre- and post-treatment measures of depression (i.e. 17 or 24 item HAM-D, MADRS, or BDI) were provided. Studies including median and interquartile range (IQR) were included in the qualitative review portion of this study; however, these studies were excluded from the meta-analysis calculations because they did not provide information regarding whether the data distribution was normal.

### Data analysis

The Parkinson’s disease and depression diagnostic criteria used in each study was noted. Mean and standard deviation were recorded for the various depression inventories prior to and following TMS treatment. All stimulation treatment parameters (timing, intensity, location, etc.) were recorded. Administration of concomitant medication with known anti-depressant qualities were also considered. Using Comprehensive Meta-Analysis software version 3 (BioStat Englewood, NJ), Hedges G and *p*-values were calculated for all studies that provided pre- and post-stimulation means and standard deviations for the reported depression inventories. Hedge’s G values provided information on the effect size of each individual group within the studies considered. An effect size of 0.20 was considered small, 0.50 considered medium, 0.80 considered large, and > 1.20 considered very large. The total weighted average effect size was then calculated for all of the study groups implementing rTMS treatment. All study groups that applied rTMS to the DLPFC were grouped to find overall effect size as well.

## Results

A search on the PubMed research database, using the aforementioned terms, yielded 13 studies. Seven of these publications were identified as meeting the inclusion criteria for this review: 1 multi-center double-blinded sham-controlled parallel-group study, 2 randomized double-blind sham controlled studies, 1 sham-controlled trial, 2 prospective open-label studies, and 1 retrospective study (Table 1). Six of the studies reported significant improvement for at least 30 days following treatment; however, one study reported variable improvement in depression depending on stimulation area. Two studies solely reported median scores and IQR, thus containing insufficient data for the quantitative portion of this meta-analysis [25, 26].

**Table 1:** Descriptive findings of 7 studies reporting on rTMS as a treatment for depression in PD

Reference	Type	Improvement	Number	Mean age (years)	Sessions/stimulation	Time Period (days)	Primary rTMS Site	rTMS Side	Follow-up	Depression Scales
Brys (2016) [18]	Multi-center	No	61	63	10/1000 to 2000 at 10 Hz	14	DLPFC	L	30 days	HAM-D
Dragasevic (2002) [22]	Prospective	Yes	10	60	10/100 at 0.5 Hz	10	DLPFC	B	21 days 30 days	HAM-D BDI
Epstein (2007) [23]	Prospective	Yes	14	62	20/1000 at 10 Hz	20	DLPFC	L	6 weeks	HAM-D BDI
Fregni (2004) [24]	Sham-controlled	Yes	42	65	10/40 at 15 Hz	14	DLPFC	L	2 weeks 8 weeks	HAM-D BDI
Makkos (2016) [25]	Randomized sham-controlled	Yes	46	66	10/600 at 5 Hz	10	M1	B	1 day 30 days	MADRS BDI
Pal (2010) [26]	Randomized sham-controlled	Yes	22	68	10/600 at 5 Hz	10	DLPFC	L	1 day 30 days	MADRS BDI
Torres (2015) [27]	Retrospective	Yes	45	63	14/1000 at 10 Hz^a^	21	PFC	B	30 day	HAM-D BDI

*DLPFC* dorsolateral prefrontal cortex, *M1* motor cortex, *PFC* prefrontal cortex, *L* left, *B* bilateral

^a^1000stim at 10 Hz for PFC; 900 stim at 1 Hz for M1

All seven studies measured depression pre- and post-treatment via one or more of the following depression inventories: HAM-D, MADRS, or BDI. Number of treatments, time period of treatments, stimulation location, and follow-up timing varied across studies. Table 2 and Table 3 show that, with one exception, all studies reported a large effect size and significant *p*-values [18]. Additionally, total weighted average effect size was calculated at 1.32 across all study groups that applied rTMS. The weighted average effect size of study groups that applied the treatment to the DLPFC only was 1.37.

**Table 2:** Effect size of studies reporting mean changes in HAM-D. Epstein et al. (2007) used the 17 and 21 item HAM-D inventory, noted under “Treatment.” Post-treatment population sizes (*n*) that changed are noted under corresponding studies groups by parentheses

Reference	Treatment	Number	Hedge’s G	*p*-value
Brys (2016) [18]	M1 Stim + DLPFC Stim	20 (19)	0.698	0.031
Brys (2016) [18]	M1 Stim + DLPFC Sham	14	1.287	< 0.001
Brys (2016) [18]	M1 Sham + DLPFC Stim	12	0.185	0.495
Brys (2016) [18]	M1 Sham + DLPFC Sham	15	1.559	< 0.001
Dragasevic (2002) [22]	DLPFC Stim	10	1.377	0.001
Epstein (2007) [23]	DLPFC Stim (17)	14 (8)	1.581	0.001
Epstein (2007) [23]	DLPFC Stim (21)	14 (8)	1.686	0.001
Fregni (2004) [24]	DLPFC Stim + Medication Sham	21	2.103	< 0.001
Fregni (2004) [24]	DLPFC Sham + Medication	21	2.012	< 0.001
Torres (2015) [27]	PFC Stim	16	2.897	< 0.001

**Table 3:** Effect size of studies reporting mean changes in BDI

Reference	Treatment	Number	Hedge’s G	*p*-value
Dragasevic (2002) [22]	DLPFC Stim	10	1.261	0.002
Fregni (2004) [24]	DLPFC Stim + Medication Sham	21	1.729	< 0.001
Fregni (2004) [24]	DLPFC Sham + Medication	21	1.843	< 0.001
Torres (2015) [27]	PFC Stim	16	1.712	< 0.001

## Discussion

This meta-analysis sought to evaluate the efficacy of rTMS to alleviate depression in patients with PD. Of 7 studies meeting the inclusion criteria, 5 contained sufficient data on depression (HAM-D and/or BDI) to perform quantitative evaluations. Because both inventories were used across the 5 studies, evaluation of rTMS from both clinician and patient perspectives was possible. Apart from Brys and colleagues (2016), all studies reported rTMS to be highly effective in improving depression (Hedge’s G > 1.2). To bolster these findings, individual *p*-values show that this trend was not observed by chance (*p* <  0.05). When all study groups implementing rTMS were combined, high efficacy was observed by both patients and clinicians. Combining study groups that specifically examined rTMS to the DLPFC yielded an even higher treatment efficacy. Results of this meta-analysis confirm that rTMS significantly lowers both the self-reported BDI and the health care provider-determined HAM-D. Ultimately, these findings suggest that rTMS may be an effective treatment for depression in patients with PD.

### Stimulation sites

Three studies report significant findings when administering rTMS to specifically the left DLPFC, the location most commonly targeted when treating depression in the non-Parkinson’s population [23, 24, 26]. An open study by Epstein and colleagues directly sought to examine the treatment’s effects in an inpatient setting [23]. The study yielded improvement at post-treatment visits (3 days-post) as well as at 3–6 week follow-up. Our results echoed these findings, showing an extremely notable relationship with high significance between treatment and improved HAM-D and BDI scores (Tables 2 and 3). In a sham-controlled trial, Fregni et al. compare treatment of depression with medication to treatment with magnetic stimulation [24]. The authors found that treatment with rTMS is equally as effective as fluoxetine in treating depression and in most cases better tolerated. In both self-report and clinician-determined measures, very large effect sizes with high significance were found for the medication and stimulation groups, showing equally notable effects on depression (Tables 2 and 3). Similarly, Pal and colleagues use a randomized, double-blind, placebo-controlled study to evaluate rTMS to the left DLPFC. In both short term and long term follow-up evaluations (MADRS and BDI), patients reported significant improvement in depressive symptoms. Interestingly, long term follow-ups (at 30 days) yield a larger significant improvement in depression scores than the immediate (1 day-post) follow-up. Although effect size could not be evaluated due to insufficient data, the authors provide evidence that patients with PD who experience mild to moderate depression may respond favorable to rTMS.

Two studies reported on the effects of bilateral prefrontal cortex stimulation. In an open study, Dragasevic and colleagues studied bilateral rTMS to the prefrontal cortex [22]. The authors found that the HAM-D and BDI improved significantly upon immediate follow-up, as well as 21 and 30 days post-treatment. These results are strengthened by a significant and noteworthy effect size post-treatment (Tables 2 and 3). In a retrospective study, Torres and coworkers identify the effects of TMS on various symptoms experienced by patients with PD, including depression [27]. Significant improvement was seen in HAM-D and BDI scores from before to after, and extended to follow-up evaluation. As seen with the previous studies, a high effect size and significance further illustrate these reports (Tables 2 and 3). Torres et al. once again demonstrate the efficacy of TMS as a treatment for Parkinson’s patients with depression.

Unlike the previously discussed studies, Makkos and colleagues solely tested for improvement in depression with stimulation to the motor cortex [25]. In a randomized, double-blind, placebo-controlled project, the authors applied bilateral rTMS to the M1 cortical region of patients. According to the MADRS and the PDQ-39, depression severity and health related quality of life improved significantly for the treatment group. As with the study by Pal and colleagues, insufficient data prevent an effect size analysis.

### Alternative findings

Among the studies considered in this review, the most common location of TMS stimulation was the left DLPFC; however, bilateral DLPFC and M1 stimulation was also reported (Table 1). In their multi-center trial, Brys and colleagues examined potential effects of rTMS to the motor cortex (M1) and left DLPFC on both motor functioning and mood [18]. Among the 50 patients who completed all study visits, the authors found that stimulation to M1 yielded improved motor function; however, unexpectedly, stimulation to the left DLPFC yielded no significant changes in the depression 1 month after treatment. When stimulation of M1 and the left DLPFC were done together, no synergistic effect was observed. The authors purport that while stimulation did not appear to effectively treat patient mood, a longer duration of treatment may yield different results.

Effect sizes and *p*-values calculated for each experimental group in this study mirrored the contradictory findings reported by Brys and colleagues (Tables 2 and 3). With regard to M1 stimulation alone, a very large effect size was calculated; this suggests that M1 rTMS stimulation reduces depression (*p* <  0.05). Alternatively, little to no effect was observed following stimulation to the DLPFC alone, raising some doubt about the efficacy of rTMS. However, when further evaluated, the result was found to have no significance (*p* = 0.495), challenging the reliability of the finding. When both M1 and DLPFC were stimulated together a moderate effect size was observed. Full sham treatment yielded high effect sizes. Together these results seem to indicate that three of the experimental groups appear to have some effect on reducing depression: M1 stimulation, simultaneous M1 and DLPFC stimulation, and full placebo stimulation.

### Total weighted average effect size

Despite the contradictory findings by Brys and coworkers, calculation of total weighted average effect size helped to resolve any discrepancy between the study results. When all study groups that applied rTMS stimulation were considered together and weighted based on sample size, effect size (1.32) overwhelmingly suggested a notable reduction in depression following rTMS. Further, among all study groups applying rTMS to the DLPFC specifically an even more notable effect was found (1.37). These weighted overall calculations serve to remedy any concern regarding the contradictory study.

Despite these findings, it is important to recognize that only one of the studies in the quantitative analysis used a true sham treatment as a control and subsequently reported a strong placebo effect (Hedge’s G > 1.2; *p* <  0.05) [18]. A major limitation for all treatments of depression is the high placebo effect. We acknowledge that the findings reported in this meta-analysis could be a result of placebo. Future studies, must incorporate reliable control groups in order to examine a potential placebo effect. Along these lines, experimental design and stimulation parameters varied greatly across all studies. New research must begin to establish standards for variables such as stimulation frequency and location. To this end, it is of note that stimulation parameters in Table 1 vary considerably between papers with the same target and standardization of these parameters may be difficult due to individual demographic variability. Provided that rTMS treatment parameters can be standardized in this way, our findings bring light to a promising alternative anti-depressant therapy. As examined from both clinician and patient perspectives, this meta-analysis strongly suggests that rTMS can relieve clinical depression in patients with Parkinson’s disease.

## Conclusions

This meta-analysis qualitatively and quantitatively evaluated the use of rTMS for the treatment of refractory depression in patients with PD. Individual study groups and overall effect sizes yielded promising reductions in self-reported and clinician-administered inventories. These findings encourage further exploration of rTMS as an alternative to traditional anti-depressant pharmacology in the Parkinson’s population.

## Abbreviations

BDI: Beck Depression Inventory
DLPFC: Dorsolateral prefrontal cortex
HAM-D: Hamilton Depression Inventory
IQR: Interquartile range
MADRS: Montgomery-Asberg Depression Rating Scale
PD: Parkinson’s disease
rTMS: Repetitive transcranial magnetic stimulation
